# Understanding Myths in Pregnancy and Childbirth and the Potential Adverse Consequences: A Systematic Review

**DOI:** 10.21315/mjms2019.26.4.3

**Published:** 2019-08-29

**Authors:** Norain Ahmad, Sharifah Fazlinda Syed Nor, Faiz Daud

**Affiliations:** Department of Community Health, Faculty of Medicine, Universiti Kebangsaan Malaysia, Kuala Lumpur, Malaysia

**Keywords:** culture, perception, pregnancy, parturition, complications

## Abstract

The trend of choosing natural birth at home without proper supervision is gaining more attention and popularity in Malaysia. This is partly due to wrong beliefs of modern medical care. It prompts the need to explore further into other myths and wrong beliefs present in communities around the world surrounding pregnancy and childbirth that may lead to harmful consequences. A total of 25 literatures were selected and reviewed. The most reported wrong belief is the eating behaviour such as avoiding certain nutritious fruits besides eating saffron to produce fairer skinned babies which in fact contains high doses of saffron that may lead to miscarriage. The most worrying myth however, is that unregulated birth attendants such as doulas have the necessary knowledge and skills to manage complications in labour which may well end up in perinatal or even maternal death. Other myths suggested that modern medical care such as vaginal examinations and baby’s heart monitoring in labour as unnecessary. A well-enforced health education programme by well-trained healthcare personnel besides sufficient number of antenatal care visits are needed to overcome these myths, wrong beliefs and practices. In conclusion, potential harmful beliefs and practices in pregnancy and childbirth are still abound in today’s communities, not just in least developed and developing countries but also in developed countries. Women and children are two very vulnerable groups, therefore debunking myths and eliminating harmful practices should be one of a healthcare provider priority especially those in the primary care settings as they are the closest to the community.

## Introduction

In February 2018, Malaysia was shocked by a heart-breaking news of a stillborn baby in the southern part of the country. Investigations into the death of the baby by the health authorities revealed that it was an outcome of a complicated homebirth, unfortunately this was not the first case. Since 2013, there were a number of cases of maternal and perinatal morbidity and mortality related to inappropriate homebirth. Despite continuous effort by the government to give the best medical and health services to the population, the trend of choosing natural birth at home without proper supervision which first originated in the West is gaining more attention and popularity in Malaysia.

Zielinski et al. (1) summarised in their study that homebirth rates in Western countries ranged from 0.1% in Sweden and up to 20% in Netherlands. Home birth became a favourable choice for pregnant women due to reasons such as women’s right to choose their preferable environment, wanting to avoid medical intervention, e.g. episiotomy, induction of labour etc. and disagreement between mother and healthcare provider regarding birth plan (1). On the other hand, poor maternal and perinatal outcomes such as anaemia in pregnancy and low birth weight among new-borns are still significant now where food and healthcare are generally available and accessible. This similar situation is unfortunately seen in Malaysia as well.

Malaysian healthcare services is listed as one of the best in the world proven by the improvement in Maternal Mortality Ratio (MMR) trend since post-independence from 250 deaths per 100,000 live births to 28 deaths per 100,000 live births in 2010 (2). This remarkable pattern of MMR is the result of few main factors, such as cooperation and the commitment of the government to improve maternal and child health; feasibility and accessibility of maternal and child health services; improving family planning education and information and increasing access to healthcare facilities (3). Unfortunately, in 2012 the MMR adjusted to place of delivery was 296.4 deaths in 100,000 live births for homebirth compared to 19.5 and 18.6 for government and public health facilities, respectively.

There are many reasons as to why these women chose to deliver at home without a trained midwife supervision. However, based on the Theory of Reasoned Action, it is hypothesised that behaviour and action originated from beliefs mainly of the final outcome. Therefore, if these people knew that what they believe may lead to poor maternal or perinatal outcomes, they will less likely to remain steadfast to the beliefs and the behaviour related to it. This theory can be applied to health education programme by the health authorities in countering myths and wrong beliefs that may have been the norms for generations.

Most parts of the world have their own beliefs and practices in pregnancy that sometimes make the practices to be similar or different from one another. As reported by the United Nations, Convention on the Elimination of All Forms of Discrimination against Women, there are few cultural practices or beliefs that can affect women’s health such as nutritional taboo during pregnancy that can cause malnutrition. Besides that, birth practice with traditional attendants is also a concern as traditional birth attendants are not trained in managing emergency situations (4).

Those are reported beliefs and practices known to the health authorities. Could this be the tip on the iceberg? What are other myths and pregnancy beliefs that can affect health negatively which we are unaware of? To answer these questions, a systematic review was done to identify the presence of myths and wrong beliefs in pregnancy and childbirth globally and to discuss the potential adverse outcomes related to them. The findings of this study will hopefully benefit policy makers and healthcare providers in understanding the current maternal and childbirth issues for future health programmes planning.

## Materials and Methods

The search process for the articles was done from 15 Feb 2018 until 26 Mar 2018. We used four search engines; Google Scholar, Ovid, Scopus and PubMed. We used PRISMA checklist for the workflow of this systematic research. The keywords used were: i) myths in pregnancy, ii) pregnancy myths, iii) wrong beliefs in pregnancy, iv) home birth, v) lotus birth, vi) natural birth, vii) gentle birth, viii) hypnobirthing, ix) waterbirth, x) traditional practice in pregnancy xi) culture in pregnancy, and xii) perception in pregnancy.

**Figure 1:**
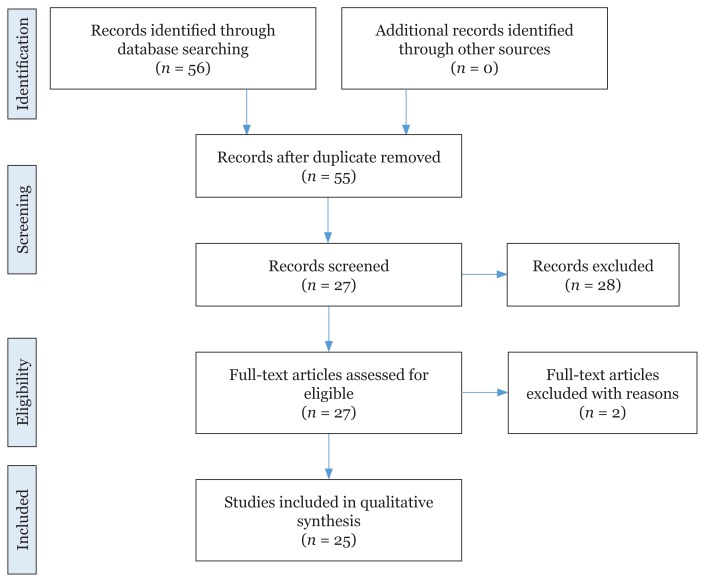
PRISMA flowchart of article selection

We chose all articles discussing myths or beliefs in pregnancy and childbirth worldwide that were published between Jan 2013 and Dec 2017. There were 56 peer-reviewed articles identified from the four databases and after removing duplicated articles, the total articles for screening became 55. Next, based on the exclusion criteria whereby articles that discussed myths that have no potential effects on health were excluded. Subsequently, the eligibility of the articles was evaluated based on the methodology especially on the validity of the study design. Therefore, the total final literatures reviewed in this study came to 25 journal articles (Figure 1). To ensure the understanding of contents of the articles, all 25 articles were read and reviewed twice, initially by the first reviewer, then the second. We came out with a summary of the characteristics of the articles selected based on the authors, year of publication, study population, study design and tools as well as the outcome measures.

## Results

This systematic review included 25 journal articles published from 2013 to 2017. The journal articles included two articles each from India, USA, Australia, Kenya, Nigeria, Malaysia and Ethiopia. One article each from Nepal, Tanzania, Pakistan, Philippines, Italy, Iran, Israel, Ghana, Bangladesh, Turkey and Laos. Nine of the studies used cross sectional design and the rest of the journal articles were qualitative studies. The studies included mostly used questionnaires, focused group discussions and in-depth interviews.

Table 1 outlined the characteristics of the journal articles in this review. A thematic analysis was done to all the myths found in the literatures reviewed, all of them can be categorised into these main themes; beliefs in eating behavior, beliefs in physical activity, beliefs in birthing process, beliefs in birth attendants and beliefs in placenta and umbilical cord.

### Beliefs in Eating Behaviour

The most discussed about pregnancy and child birthing myths in the world belong to the eating behaviour theme. Thirteen of the articles reviewed involved myths in this category. Parmar et al. (5) in India reported that 31.7% of respondents believed that eating saffron results in fairer skin of the child. While saffron has its own beneficial medicinal effect, exposure to very high doses of saffron may increase the risk of miscarriage due to its uterotonic properties (6). At higher doses saffron has also been shown to cause embryonic malformation in animal’s models and is therefore suggested to be avoided by pregnant women (6).

Certain fruits and vegetables are often believed to be bad for consumption in pregnancy. Two papers from India and one literature from Malaysia reported that papaya, jackfruit, bitter gourd and pineapple are among the fruits and vegetables believed to cause miscarriages (5, 7, 8). One literature from Kenya reported that eating green leafy vegetables can cause hiccups and gasping in babies during breastfeeding (9). Whereas in Italy, sugar is believed to be the cure for dizziness and weakness during pregnancy (10). In a study of dietary patterns and development of gestational diabetes mellitus (GDM), Shin et al. (11) reported that higher consumption of refined grains, fat, added sugar and low intake of fruits and vegetables imposed higher odds for developing GDM.

The Maasai women in Tanzania and Ghana generally restrict food in pregnancy to avoid having bigger babies and difficult labour (12, 13) whilst women in Shashemene in Ethiopia avoid nutritious food such as linseed, honey, milk and yoghurt (14). Whereas women in southwest Nigeria consumed herbal concoction to have smaller babies and ease delivery (15). Women in Turkey avoid fish for fear of having fish-mouthed babies and liver for fear of having babies with stained skin (16). Women in south-eastern Nigeria and a rural district in Kenya avoided snails, bushmeat and eggs which were their main daily source of protein also due to fear of big babies and prolonged labour (17, 18). As a result, these women take very little food on top of their pre-existing nausea and loss of appetite. They are often left feeling tired and malnourished and subsequently risk having low birth weight babies due to insufficient calorie intake. Choudhary et al. (19) reported that daily consumption of less than 2000 kcal is associated with higher risk for low birth weight babies.

On the other hand, a common myth in pregnancy that may have an opposite effect which is eating for two. This was reported in the papers from Philippines and Italy (10, 20). Maternal obesity and increased weight gain during pregnancy that may result from this particular myth are found to be associated with GDM, gestational hypertension, pre-eclampsia, large for gestational age babies and childhood obesity (21). It is also a known risk factor for deep vein thrombosis which can be fatal due to pulmonary embolism.

### Beliefs in Physical Activity

Three of the papers reviewed reported myths involving physical activity during pregnancy. Marshall et al. (22) reported that in a rural community in south-eastern USA, physical activity among pregnant women is generally not undertaken due to the belief that daily life activities provide sufficient exercises. Some thought that rest is more important than physical activity, while others believed that physical activity poses safety risks to both mother and baby (22). Unlike pregnant women of Maasai in Tanzania, they will steadily increase their workload of daily chores through the second and third trimester up to the point of exhaustion and miss their antenatal check-ups, in preparation for their postnatal period in which they will stay in their homes for three months (12). Whereas in Pakistan, Atif et al. (23) reported that 76.4% of respondents believed that mild to moderate weight lifting causes miscarriage.

Center for Disease Control and Prevention (CDC) stated that physical activity is good for overall health of pregnant and postpartum women since it also improves mood in the postpartum period. The recommended amount of physical activity by the 2008 Physical Activity Guidelines for American adults is at least 150 min per week of moderate-intensity exercises such as brisk walking during and after pregnancy in which it is best to spread the activity throughout the week whereas healthy women who already do vigorous-intensity activity can continue doing so as long as they stay healthy throughout their pregnancy (24). It does not pose any safety risks neither does it causes low birth weight babies, early delivery or miscarriage. It is a known fact that immobility is one of the main risk factors for deep vein thrombosis, therefore pregnant women are encouraged to be as physically active.

### Beliefs in Birthing Process

Eleven of the reviewed literatures discussed about beliefs that fall under the birthing process theme. Three of the papers reported were from Australia, USA and Israel that stated that birth is a natural event and modern medical care during delivery interrupts natural birth process (25–27). In addition, respondents who preferred natural birth at home perceived modern medical care and interventions such as vaginal examinations, baby’s heart monitoring, injections and painkillers as unnecessary (25). Women in south central Ethiopia avoided hospital delivery for fear of having minor or major operations such as caesarean sections (28). Whereas women in rural Laos and southwest Nigeria believed that attending antenatal care and giving birth at a health facility is needed only when they felt that they are not healthy (15, 29). Some papers reported that modern medical care during delivery is seen as disrespecting and disempowering of women in labour (25, 27, 30). It was also reported that some people placed comfort as their top priority and often relate comfort to safety (25, 30). This belief is a concern since even among low risk pregnant women who have been assessed and allowed to deliver at home under a midwife supervision, 40% of them ended up being referred to hospital due to unexpected complications in labour (31).

In Israel, there are two main beliefs about the birthing process; belief that birth is a medical process and belief that birth is a natural process (26). Those who believed birth is a natural process had similar ideation about natural birth as in the rest of the world that it is empowering of women and should be allowed to proceed at its own pace (26). In a case control study of intrapartum stillbirth and livebirth in Nepal, it was found that there was an increased risk of intrapartum stillbirth when fetal heart rate was inadequately monitored and when the progress of labour was not monitored using a partogram (32). Therefore, if labour is allowed to progress at its own pace without any intervention, there is an increased likelihood of having poor perinatal outcomes. In contrast, a paper from Malaysia reported that most respondents used herbal medicine to facilitate labour (33) which may risk precipitating labour and postpartum haemorrhage.

In regards to mode of delivery, one paper from Pakistan reported that respondents preferred vaginal delivery over caesarean section due to the belief that pregnancy weight gain persists longer after caesarean section (23). This may not be totally true as weight loss after delivery is much more related to the amount of physical activity and most importantly the dietary pattern practiced by these women. Exclusive breastfeeding is also found to increase weight loss post-delivery (34). On the other hand in Iran, the mode of delivery can be perceived as a status symbol, whereby normal vaginal delivery is seen as a low cost mode of delivery whereas caesarean section is preferred as it is seen as a prestigious mode of delivery (35). This is in alignment with the commonly used expression ‘too posh to push’. It may cause an increased rate of unnecessary caesarean sections which may indirectly increase the incidence of maternal morbidity and mortality. In a Swedish case control study, Karlström et al. (36) reported that maternal complications occurred more frequently among women undergoing caesarean section. Whereas in Brazil, it was found that the risk of postpartum maternal death was almost three-fold higher with caesarean than vaginal delivery (37). This proves that caesarean section is not superior to normal vaginal delivery unless indicated.

### Beliefs in Birth Attendants

Three articles were found to discuss about beliefs in birth attendants. A paper from Australia reported on reasons why women chose unregulated birth worker over certified trained midwives. These women believe unregulated birth worker provides the best of both worlds which is support for physiological paradigm of birth and birth care that made them feel safe (25). For them, births at home by registered midwives is an introduction of hospital policies, rules and regulation into women’s homes which are very strict and rigid (25). Healthcare workers in health facilities were perceived to be not welcoming towards them (28). Some also believed that a doula or traditional birth attendant has the necessary knowledge and skills to anticipate and manage complications in labour (25, 28, 30). This is a wrong perception because they are not trained to perform clinical and medical tasks therefore would not know how to handle complications.

Most obstetric complications could only be prevented or managed if women had access to a skilled birth attendant during childbirth. Increment of coverage of skilled birth attendants during delivery may have contributed to the decline of maternal mortality from 1990 to 2015 globally according to WHO (38), especially in Sri Lanka, Malaysia, Thailand, Egypt, Honduras and Bangladesh (39).

### Beliefs in Placenta and Umbilical Cord

There were four papers discussing about beliefs in placenta and umbilical cord. A paper from Australia found that respondents chose to leave the placenta intact (lotus birth) after delivery because they believed that lotus birth is the baby’s right and cutting the cord is seen as to take control over something that is not yours (40). They also believed that there is a spiritual connection between the baby and the placenta (40). Royal College of Obstetrics and Gynaecologists (RCOG) does not recommend umbilical non-severance or lotus birth because there is a risk of infection spreading to a baby since the placenta contains blood (41). Besides the practice of lotus birth, Burns (40) reported that some of the respondents consumed their placenta because they believed that it has beneficial medicinal properties. A systematic review regarding placentophagy concluded there is no strong evidence of the beneficial effect of consuming placenta and further studies are needed especially on the health effects of placentophagy on humans (42).

Respondents from Nepal and Laos practiced cutting the umbilical cord with a bamboo, razor or sickle and then applying cooking oil, ghee, toothpaste or ash (29, 43). Cutting the cord with a dirty apparatus such as a razor blade or sickle increases the likelihood of contracting neonatal tetanus in which WHO estimated 34,019 new-borns died from neonatal tetanus globally in 2015 (44). Another belief related to placenta is that inserting a piece of cloth into women’s mouth to induce vomiting (gag reflex) or blowing air into a bottle can expel placenta (13, 43). Retained placenta is an obstetrical emergency that needs urgent medical attention. If it is not treated properly the woman may suffer from postpartum haemorrhage and subsequently death.

## Discussion

Cultural beliefs and practices in pregnancy and childbirth are still relevant in communities today especially in the rural setting. It encompasses many aspects of pregnant women’s daily life activities from eating behaviour up to the process and mode of delivery. As social media has become significant in most parts of the world, these myths and wrong beliefs can easily be shared and spread to other parts of the world. However not all these beliefs are culturally related in fact, some of these beliefs are due to peer pressure, poor knowledge or ignorance. Whichever way it is, this issue can be overcome by implementing a well enforced health education programme which should be instated early and well throughout the course of a pregnancy.

WHO encourages early antenatal booking and sufficient antenatal visits from then on. Previously, the recommended number of antenatal visits is at least four times before delivery. However in 2016, it was changed to eight visits in which the first contact should be at 12 weeks then the subsequent visits will be at 20, 26, 30, 34, 36, 38 and 40 weeks’ gestation (45). Higher frequency of antenatal contacts can increase the opportunity for healthcare providers to build good rapport and give sufficient advice and valuable knowledge that may guide these women in making the right decisions in pregnancy and childbirth for example in choosing the right food to consume or avoid as well as choosing the best place or mode of delivery.

Healthcare personnel on the other hand should have the right attitude, skills and knowledge to educate these women as soft skills is one of the main areas that is often lacking among healthcare workers. As a result, these women are easily influenced by their peers and family members more than the medical experts themselves. In addition, healthcare providers should also be accommodating by involving pregnant women especially in coming up with the agreed birth plan as much as they can. However, to end unsupervised homebirth, supervised natural birth services may need to be established in our healthcare facilities with proper guidelines and standard operating procedures in place. Besides that, there should be adequate resources and training of staffs before introducing this new policy.

## Conclusion

From this systematic review, it was found that myths and wrong beliefs in pregnancy and childbirth still exist not just in the least developed or developing countries, but also in the developed countries all around the world. Women and children are two very vulnerable groups in the community and ensuring the wellbeing of these groups have always been the main agenda for many health organisations in the world. Due to this fact, debunking myths and eliminating practices that may have potential adverse health effects in these groups should be one of a healthcare provider priority especially those in the primary care settings as they are the front liners closest to the community.

## Figures and Tables

**Table 1:** Characteristics of included studies

No.	First author	Study Population	Study Design/Tools	Outcome
1.	Parmar et al. (5)	Women above 18 years old in rural Khodu, Surendranagar, India	Cross-sectional study, questionnaire	Taboos and misconceptions in pregnancy
2.	Sharma et al. (43)	Women with recent pregnancy and/or with a child under the age of 2, their mother-in-laws, husbands and health service providers in Nepal	Qualitative, in-depth interview, focused group discussion	Beliefs around childbirth
3.	Lennox et al. (12)	Pregnant women in Masaai, northern Tanzania	Qualitative, semi-structured individual interview, 24 h diet recall	Dietary pattern and nutrition in pregnancy
4.	Marshall et al. (22)	Pregnant women in rural community of southeast USA	Qualitative, questionnaire	Barriers to physical activity in pregnancy
5.	Burns (40)	Pregnant women planning for home birth or women who gave birth at home in the last 3 years in Victoria, Australia	Qualitative, in-depth interviews	Placenta beliefs and rituals
6.	Atif et al. (23)	Patients who attended Combined Military Hospital, Peshawar Pakistan	Cross-sectional study, questionnaire	Prevalence of myths in reproductive age women
7.	Rigg et al. (25)	Women and unregulated birth workers in Australia	Qualitative, in-depth interviews	Perception of women choosing unregulated birth worker
8.	Bermio, Reotutar (20)	Multigravida women of Ilocos Sur District Hospital, Phillipines	Cross-sectional study, questionnaire	High extend of cultural beliefs and practices among the women
9.	Bernhard et al. (27)	Pregnant women in doula class in western Michigan, USA	Qualitative study, focused group discussion	Perception of current delivery intervention and beliefs in birthing mechanism
10.	Guggino et al. (10)	First trimester pregnant mother admitted to St. Anna Hospital of Turin, Italy	Cross-sectional study, semi-quantitative food-frequency questionnaire	Myths in nutritional intake in pregnancy
11.	Roudsari et al. (35)	Pregnant women, women with previous childbirth experience, midwives, obstetricians and non pregnant women in Tonekabon, northern of Iran	Qualitative study-focused, ethnographic semi-structured interviews and participant observations	Myths and wrong beliefs regarding birth process
12.	Gogoi (8)	Bodo community of Nic-Chinakona, Assam, India	Qualitative-ethnographic study, interviews	Myths and food taboos in pregnancy
13.	Preis, Benyamini (26)	Pregnant women in Tel Aviv, Israel	Quantitative and qualitative study, birth belief scale-questionnaire	Beliefs in birth process
14.	Ezeama, Ezeamah et al. (9)	Women who had delivered babies a year prior in Akin Yele LGA, Oyo State Nigeria	Quantitaive and qualitative study, questionnaire and focused group discussion	Atitude and sociocultural pactice during pregnancy
15.	Otoo et al. (13)	Pregnant women and women who delivered within 12 months prior in Shama district of the western region of Ghana	Qualitative, focussed group discussion and in-depth interview	Traditional practices associated with pregnancy and childbirth
16.	Sarker et al. (30)	Stakeholders, healthcare workers and women in Sunamganj district of Bangladesh	Qualitative cross-sectional, in-depth interview	Preference of home delivery with traditional birth attendants and the associated factors
17.	Riang’a et al. (17)	Pregnant and postnatal women in Kalenjin, Kenya	Qualitative, semi-structured interview	Restricted and recommended food in pregnancy
18.	Ekwochi et al. (18)	Women who had carried at least one pregnancy to term in Enugu south east Nigeria	Qualitative cross-sectional, questionnaire	Food avoidance and the associated factors
19.	Taşçı-Duran, Sevil (16)	Pregnant women (15–49 years old) in Bornova Izmir, Turkey	Qualitative ethnonursing, in-depth face-to-face semi-structured interview	Health behaviours during prenatal period
20.	Mohamad, Chong (7)	Malay pregnant women in Kuala Lumpur, Malaysia	Cross-sectional, face-to-face questionnaire	Prevalence and types of food taboos and its reason for avoidance
21.	Zepro (14)	Pregnant women in Shashemene district of Ethiopia	Cross-sectional questionnaire	Prevalence of taboos/misconceptions in pregnancy and its associated factors
22.	Law, Soon (33)	Antenatal and postnatal mothers admitted to Hospital Universiti Sains Malaysia, Kelantan, Malaysia	Cross-sectional interview, structured questionnaire	Prevalence and description of herbal medicine usage in pregnancy
23.	Roro et al. (28)	Women and their men/partners who delivered past 2 years in Butajira, Ethiopia	Qualitative, focussed group discussion	Reasons for not choosing to deliver at health facilities
24.	Sychareun et al. (29)	Postnatal women, husbands, mothers, traditional birth attendants, head villagers, Lao Women’s Union members and healthcare workers in central Laos	Qualitative interview, focussed group discussion	Cultural beliefs and practices surrounding pregnancy, antenatal and postpartum care
25.	Okafor et al. (15)	Women (15–45 years old) who delivered 2 years prior to study in southwest Nigeria	Qualitative, focussed group discussion	Determinants and reasons of choosing orthodox versus unorthodox care
